# Microbial community development of biofilm in Amaranth decolourization technology analysed by FISH

**DOI:** 10.1080/13102818.2014.947725

**Published:** 2014-10-21

**Authors:** Mihaela Belouhova, Irina Schneider, Stoyan Chakarov, Iliana Ivanova, Yana Topalova

**Affiliations:** ^a^ Faculty of Biology, Sofia University ‘St. Kliment Ohridski’, Sofia, Bulgaria

**Keywords:** wastewater treatment, Amaranth, non-culturable bacteria, *Pseudomonas* sp., fluorescent in-situ hybridization, technological parameters

## Abstract

The aim of this study was to elucidate the role, the space distribution and the relationships of the bacteria from the genus *Pseudomonas* in a biofilm community during semi-continuous Amaranth decolourization process in model sand biofilters. The examined parameters of the process were as follows: technological parameters; key enzyme activities (azoreductase, succinate dehydrogenase, catechol-1,2-dioxygenase, catechol-2,3-dioxygenase); the number of azo-degrading bacteria and the bacteria from genus *Pseudomonas* (plate count technique); the amount and the location of *Pseudomonas* sp. using fluorescent in situ hybridization (FISH).

The results showed that the increase of the Amaranth removal rate with 120% was accompanied with increase of the enzyme activities of the biofilm (azoreductase activity – with 25.90% and succinate dehydrogenase – with 10.61%). The enzyme assays showed absence of activity for сatechol-1,2-dioxygenase and catechol-2,3-dioxygenase at the early phase and high activities of the same oxygenases at the late phase (2.76 and 1.74 μmol/min mg protein, respectively). In the beginning of the process (0–191 h), the number of the culturable microorganisms from genus *Pseudomonas* was increased with 48.76% but at the late phase (191–455 h) they were decreased with 15.25% while the quantity of the non-culturable bacteria from this genus with synergetic relationships was increased with 23.26%.

The dominant microbial factors were identified in the structure of the biofilm during the azo-degradation process by using FISH analysis. Furthermore, the inner mechanisms for increase of the rate and the range of the detoxification were revealed during the complex wastewater treatment processes.

## Introduction

The key factor, accomplishing transformation of the pollutants in the wastewaters, is the highly adapted biological systems – biofilms or activated sludges with specific structure. That is why the decoding of the qualitative and quantitative microbial structure of highly effective biological systems is from significant importance for the regulation and management of the water treatment processes.[1]

This rule is valid for all water treatment technologies, but the governing of the microbial micro- and macrostructure of the biofilms, adapted to the biodegradation of hazardous xenobiotics, possess the potential to increase the effectiveness of the detoxification process.[1,3] The answers to the questions ‘Who are the dominant genera of microorganisms, adapted to the detoxification?’, ‘What are their relationships with other microbial components in the microenvironments of the biofilm?’ and ‘How the changes in microbial structure lead to the changes in the biodegradation activity?’ are essential for the elucidating and application of the potential.

From another point of view it is important to reveal and construct research approaches that are the essential algorithms for elucidation and regulation of the biodetoxification potential. The classical methods for investigation of the microbial communities are based mainly on the plate count techniques. These techniques ensure the evaluation of the amount of culturable bacteria, but are not able to assess the microorganisms, that play the main biodegradation role in syntrophy and complicated co-metabolic relationships – so-called unculturable bacteria.[4,5]

The ascertained fact is that a significant part of the active xenobiotic-biodegradants participate in the syntrophic and synergetic microbial communities and their activity can be assessed by means of enzyme indicators, as well as by measuring the rate of biodegradation.[6,3] Only with the purposely constructed combination of methods (microbiological, enzymological and technological) the real catabolic potential of the biofilms functioning in the water detoxification technologies can be elucidated.

In the various investigations, it has been ascertained that the bacteria from genus *Pseudomonas* play an important role in the adaptation of biofilms for biodegradation of aryl-containing xenobiotics.[3,6–8] Therefore, a very important question is what are the amounts, the co-metabolic relationships and the specific localization of *Pseudomonas* in the biofilms that are effective in the xenobiotics elimination. The answer can be given by application of FISH (fluoresce in-situ hybridization technique).[4,5,7–10] FISH makes it possible to analyse the amount, distribution and location of *Pseudomonas* during the development of the biofilm. The diagnostics was made during the adaptation of biological system and the improvement of the water treatment technology.

A simultaneous application of different methods (plate count, FISH, enzyme activities measuring, as well as analysis of the essential technological parameters) to elucidate the role of *Pseudomonas* in the treatment process of wastewater, contaminated by azo-dye Amaranth, has been applied in this paper. This xenobiotic was chosen as a model pollutant because of the serious impact of azo-dyes on the environment.[11]

The analogous modelling of wastewater treatment technology in lab-scale sand biofilter, functioning in a semi-continuous regime, has been accomplished.

## Materials and methods

### Experimental design

**Figure 1.**
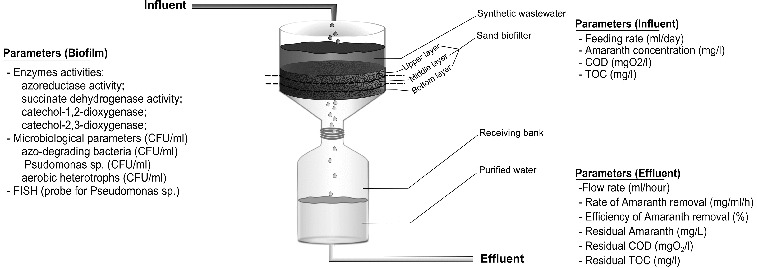
Design of the experiment and description of the parameters of the water treatment process.

Lab-scale bioreactor type ‘Sand biofilter’, operated in semi-continuous regime, has been used in the experiments (Figure 1). The biofilter had 191.7 cm^3^ of useful volume and it functioned for 20 days. To provide constant flow of wastewater through the biofilter a peristaltic pump was connected with it. The inflow of wastewater was between 520 and 792 mL/day. Chemical oxygen demand (COD) of the synthetic wastewater was 550–600 mgO_2_/L. The concentration of the organic carbon (TOC) was 230–245 mg/L. Sand layer was 30 mm high.

Gradually increasing concentration of Amaranth (10–45 mg/L) was used as an adaptation algorithm. In the course of the detoxification process two phases have been estimated – early phase and late phase. In the early phase the concentration of Amaranth was increased from 10 to 30 mg/L. The biofilm was adapted to the toxic pollutant elimination. In the late phase the inflow Amaranth concentration was increased from 30 to 45 mg/L and the biofilm developed its potential for degrading the model xenobiotic. In this phase optimal values for all technological and microbiological parameters were reached. The microbiological and enzymological parameters in the depth of the sand layer were analysed. Three layers of the biofilter with thickness of 1 cm were examined (upper, middle and bottom layer).

#### Biological system 

Activated sludge (AS) from wastewater treatment plant (WWTP) of Sofia city was used as inoculation material. The inoculation material was prepared as described in Kirilova et al.[12] Immobilization has been carried out on the basis of spontaneous adsorption and attachment of microorganisms on sand particles. Before immobilization AS was disintegrated by an ultrasonic disintegrator (3 × 10 s). In this way floculas in AS were destroyed and homogenous microbial suspension, suitable for immobilization of microbial cells, was obtained.

#### Synthetic wastewater

Salt solution (NaH_2_PO_4_ – 3.5 g/L, K_2_HPO_4_ – 5.0 g/L, (NH_4_)_2_SO_4_ – 2.5 g/L, MgSO_4_.7H_2_O – 0.3 g/L, FeSO_4_ – 0.05 mg/L, CuSO_4_ – 0.01, ZnSO_4_ – 0.005 mg/L, CoCl_2_ – 0.005 mg/L, MgCl_2_ – 0.005 mg/L, CaCl_2_ – 0.005 mg/L, Na_2_MoO_4_ – 0.005 mg/L), 3% nutritious solution (NaCl 5 g/L; peptone – 10 g/L; yeast extract 5 g/L) and definite amount of Amaranth (from 10 to 45 mg/L).

#### Quartz sand

The size was between 0.08 and 0.16 cm and the sand was kindly provided by the Bistritza Drinking Water Treatment Plant (Sofia City, Bulgaria).

#### Amaranth

The model xenobiotic was supplied by Fluka Chemical Corp. Amaranth concentration was determined spectrophotometrically (Utrospec3000, PharmaciaBiotech), λ = 520 nm.[2] The COD in water was analysed according to a standard procedure.[13] Total organic carbon (TOC) was measured with a TOC/TN analyser (TOC-V, SHIMADZU CORP., Japan).

Efficiency of Amaranth removal was calculated according to the following formula:
(1) 
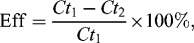
where *Ct* is the concentration of Amaranth in the moment *t*.

Rate of biodegradation was calculated according to the following formula:
(2) 
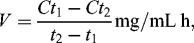
where *Ct* is the concentration of Amaranth in the moment *t*.

#### Enzymological methods

For determination of enzyme activities of the samples, cell-free extract was produced by the Feist and Hegeman [14] method modification of Topalova et al.[15]

Succinate dehydrogenase activity was determined by the method of Veeger et al.,[16] the modification of Topalova et al.[15] For studying the azoreductase activity, the method of Zimmerman et al. [17] was used in modification of Topalova.[1] Catechol 1,2-dioxygenase activity and catechol 2,3-dioxygenase activity were determined by the methods of Willets and Cain [18] and Farr and Cain.[19]

Protein content in samples was determined by the method of Kochetov,[20] using bovine serum albumin as standard.

#### Microbiological methods

The microbial diversity of the biofilm was studied by the plate count technique.[3] The azo-degraders (AzoD) were cultivated on Nutrient Agar with 50 mg/L Amaranth. The colonies with media decolourization were counted. The aerobic heterotrophs were cultivated on Nutrient Agar and bacteria from genus *Pseudomonas* were cultivated on Glutamate Starch *Pseudomonas* Agar.

#### FISH analysis

Samples from the three layers of the biofilter (upper, middle and bottom layers) were taken. The samples were fixed with 8% of paraformaldehyde. Dehydration and permeabilization were performed according Nielsen.[8] The fluorescent signal came from the 5^′^-labelled with Cy3 nucleotide probe (5^′^-GCT GGC CTA GCC TTC-3^′^).[21] As a control non-sense probe was used – NON338 (5^′^-ACT CCT ACG GGA GGC AGC-3^′^).[22] The hybridization was performed with 20% formamide. After the hybridization the samples were counterstained with DAPI (4^′^,6-diamidino-2-phenylindole) (AppliChem GmbH). The pictures were taken with fluorescent microscope Leica Microsystems DFC310FX, at 400x magnification. The digital processing of the pictures was made by using the computer program DAIME.[23] *Pseudomonas* spp. ratio to the total quantity of the microorganisms was calculated on the basis of the pictures with DAPI and the pictures with the specific probe.

## Results and discussion

The model wastewater treatment technology was studied at four levels by getting deeper in the process of the biofilm development: (1) technological level (information about the rate and the efficiency of wastewater treatment processes); (2) microbiological level (by using plate count techniques for determination of the amount of the key functional groups of microorganisms; (3) enzymological level (oxygenase-, dehydrogenase-, azoreductase activities). The obtained information served to assess the ability of the biofilm to conduct the specific biochemical reactions, which were critical for the key detoxifycation processes); (4) molecular-genetic level (the FISH provided information about the development of the biofilm and for the amount and the spatial distribution of genus *Pseudomonas* which were particularly important in xenobiotics biodegradation).

1. Technological parameters
  The following key parameters were monitored during the azo-detoxification process: flow rate, efficiency of Amaranth removal, rate of Amaranth removal, COD and TOC. For all mentioned parameters, an optimization in the late phase compared with the early phase was registered (Table 1). The efficiency and the rate of Amaranth removal were increased with 1.05% and 0.516 mg/mL h, respectively. In this time the flow rate was also increased and the biofilter treated larger volume of wastewater which was more toxic due to the higher xenobiotic concentrations during the late phase. The data showed that the biofilm was developed and was gradually adapted towards the biodegradation of the target xenobiotic – Amaranth. The data obtained for COD and TOC indicated that the biofilm was developed not only towards the elimination of the Amaranth, but also towards the increased removal of the all pollutants presented in the synthetic wastewater. The COD was decreased with 26.5 mgO_2_/L and the TOC was decreased with 6.69 mg/L.

**Table 1.** Mean values of the key technological parameters in the early and the late phase of the biofilter functioning.

Parameter	Early phase (191 h) – inflow Amaranth concentration 10–30 mg/L	Late phase (455 h) – inflow Amaranth concentration 30–45 mg/L
Flow rate (mL/h)	20.58	24.41
Efficiency of Amaranth removal (%)	92.40	93.35
Rate of Amaranth removal (mg/mL h)	0.428	0.944
COD (mgO/L)	401.21	374.71
TOC (mg/L)	114.20	107.51
2. Microbiological parameters
  In order to investigate the amount and the distribution of the key functional groups of culturable microorganisms, an analysis of the bacterial community based on plate count techniques was made. The studied groups of microorganisms were the aerobic heterotrophs, the azo-degrading bacteria and the microorganisms from genus *Pseudomonas*. The obtained data are shown in Figure 2. In the late phase of functioning, compared with the early phase, a rising in the total amount of microorganisms was found. The aerobic heterotrophs increased their numbers with 3.03%. This effect probably occurred due to the development of the biofilm during the wastewater treatment process. The data about the other two key groups of microorganisms showed the opposite tendency. The amount of the azo-degrading microorganisms decreased in the late phase with 11.35% and the amount of the microorganisms from genus *Pseudomonas* – with 15.24%. On the other hand, the reduction in the number of microorganisms was accompanied by an increase in the efficiency and in the rate of the Amaranth transformation process (Table 1) that was mainly carried out by these two groups of microorganisms. Explanation of the above-described contradiction can be found in the strengthening of the role of symbiotic, synergetic, syntrophic and co-metabolic relationships. In this way it was possible less amount microorganisms to carry out the process of biodegradation of the toxicant more effectively.[3] Our speculation was that in the biofilm, zones with synergetic active non-culturable microbial populations have been formed. Later in the study this speculation was proved.

**Figure 2.**
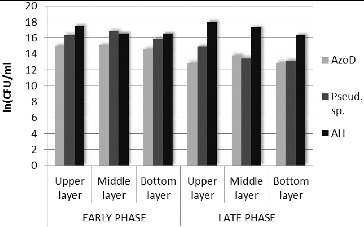
Distribution of the amount of key functional groups of microorganisms in the biofilm (azo-degrading bacteria (AzoD), aerobic heterotrophs (АН) and genus *Pseudomonas* (*Pseud*. sp.)). Data are obtained using plate count techniques.
  In the depth of the biofilm, significant differences in the distribution of the studied microorganisms were not found. The obtained data for the early and the late phase showed an increased amount of aerobic heterotrophs in the upper layer, compared with the middle and bottom layers (Figure 2). The most probable reason for this was the depletion of the nutrients in the upper layer which was in direct contact with the synthetic wastewater. It was also found that the microorganisms from genus *Pseudomonas* followed the same tendency in the late phase – they were still presented in a bigger amount in the upper layer (Figure 2). The factors supporting *Pseudomonas* growth in the upper layer were the capability of the microorganisms from this genus to degrade a large variety of xenobiotics and also the direct contact of this layer's microorganisms to the wastewater.
3. Enzymological parameters
  The enzymological status of the biofilm in two critical control points (one in the early phase – 191 h and one in the late phase – 455 h) was investigated. The obtained data confirmed the simultaneous development and specialization of a biofilm for the Amaranth biodegradation. The results from enzymological researches showed that the azoreductase (EC1.7.1.6) activity increased in the late phase with 25.90% (Table 2). This was related with the higher efficiency of Amaranth removal during the late phase (Table 1). The results for the succinate dehydrogenase (EC1.3.5.1) also demonstrated an increase during the same phase (with 10.61%). The described enzyme data indicated that the overall metabolic potential of the biofilm was expanded in the second stage of the process.

**Table 2.** Key enzyme activities for Amaranth biodegradation: azoreductase (AzoR), succinate dehydrogenase (SDH), catechol-1,2-dioxygenase (C12DO) and catechol-2,3-dioxygenase (C23DO) in the early and the late phase of the process.

	Early phase (191 h) – inflow Amaranth concentration 10–30 mg/L			Late phase (455 h) – inflow Amaranth concentration 30–45 mg/L		
Layer	Upper layer	Middle layer	Bottom layer	Upper layer	Middle layer	Bottom layer
AzoR (μmol/min mg protein)	3.47	7.98	6.57	9.33	3.36	10.00
SDH (μmol/min mg protein)	0.80	0.24	0.09	0.39	0.68	0.18
C12DO (μmol/min mg protein)	–	–	0.05	2.37	3.95	1.96
C23DO (μmol/min mg protein)	–	–	–	1.62	0.92	2.67
  The analysed enzyme indicators in our study were purposely selected. The studied enzymes catalyse the key biochemical reactions of the whole biodegradation pathway of Amaranth. In the Amaranth transformation pathway the first step is the reduction of the azo-bond (catalysed by azoreductase) and the second step is the oxidative cleavage of the aromatic rings. The second step is critical for the process of detoxification and it is catalysed by the enzymes catechol 1,2-dioxygenase (EC1.13.11.1) and catechol 2,3-dioxygenase (EC1.13.11.2). In the early phase of the functioning of the model biofilter, the above-mentioned dioxygenases had almost no activities. Unlike this, in the late phase high activity of both oxygenases was found – 2.76 μmol/min mg protein mean value for C12DO and 1.74 μmol/min mg protein mean value for C23DO.
  The obtained data matched to the metabolic logics – first the biofilm was adapted to carry out the first stages of the Amaranth biodegradation. After that, while running the processes of adaptation and development of the biofilm, the subsequent biochemical steps were unlocked. In this way, by biodegradation oriented to the stimulation and adaptation of the biofilm community, an effective and highly specialized biological system was produced. This kind of system could be used as a basis for creating a technology suitable for the real practice.
4. Molecular-genetic techniques (FISH)
  In order to follow up and prove the development of a biofilm, the application of molecular–biological approach was needed. FISH was used in order to receive the information about the availability, abundance and location of the genus *Pseudomonas* in the effective working biofilm. To perform the FISH procedure the genus *Pseudomonas* was chosen, because this genus plays the key role for the adaptation and biodegradation of aryl-containing xenobiotics.[3,6–8] The obtained data showed that a lot of the microorganisms in the biofilm belonged to the target genus, measured as an area with the specific fluorescence signal (Table 3). Significant increase of the amount of bacteria from genus *Pseudomonas* was found in the late phase, as it is seen in Table 3. This was proved by the strong fluorescent signal in the biofilm from this period (pictures with red colour in Table 3). Zones with particularly strong signal (zones with bright red colour) can be identified in the pictures. This strong signal was a result from aggregation of microorganisms in highly specialized structures, which proved the distribution of bacteria in microniches. These structures were the most probable places where the symbiotic, synergetic and co-metabolic relationships evolved and highly active microbial communities were formed. The registered by means of enzyme activities and technological indicators high biodegradation activity was related to the increased azo-biodegradation potential of the biofilm, concentrated in the newly formed microstructures.[1] The results from the FISH in fact confirmed our preliminary speculations that development of the biofilm potential towards aryl-biodegradation is connected with the increase of the specific synergetic and co-metabolic relationships that improved the processes parameters and the entire technology as well.

**Table 3.** FISH pictures of the biofilm community. Each object is shown stained with DAPI (blue colour) and with fluorescent probe specific for the genus *Pseudomonas* (red colour). The samples are taken from the three layers of the biofilter in the early (191 h) and in the late (455 h) phase of the experiment.
  In order to prove the changes in the amount of microorganisms from genus *Pseudomonas* on the basis of FISH, an additional software analysis of the images was performed.[23] The obtained data from the late phase showed an increase in the amount of the target bacteria in the upper and bottom layers (Figure 3(a)). The most probable reason for the observed effect in the upper layer was the closer contact of the microorganisms from this layer with the wastewater, e.g. the closer contact with the nutrients. The increase of the amount of the bacteria from the genus *Pseudomonas* in the bottom layer of the biofilter was favoured by the enhanced anaerobic conditions in that layer, which improved the azo-reduction of the model xenobiotic. In general, the increased amount of microorganisms from genus *Pseudomonas* corresponded to the development of the biofilm and to the adaptation of that biofilm in order to carry out the azo-detoxification process. By using FISH it was proved on genetic level that the created in the course of the technology biofilm was highly specialized and it was capable to accomplish an efficient wastewater treatment process in the conditions of xenobiotic pressure.

**Figure 3.**
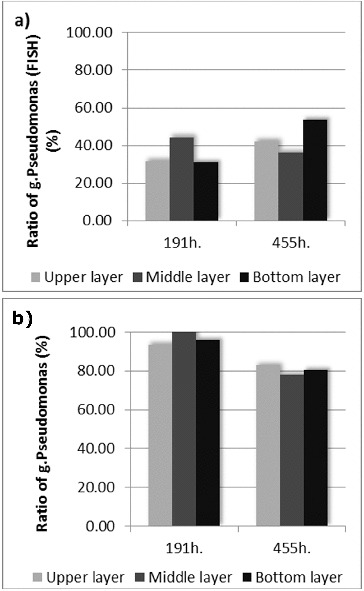
*Pseudomonas* spp. ratio to the total quantity of the microorganisms calculated on the basis of (a) FISH and (b) standard plate count techniques.
  Interesting results were obtained when the data for the amount of genus *Pseudomonas* received by means of FISH and the data for the same bacteria, obtained by plate count techniques were compared (Figure 3(a) and 3(b)). The in situ hybridization showed increase of the amount (with 23.26%) of the target microorganisms in the late phase compared with the early phase of the process. The plate count techniques showed the opposite results – decrease of the amount (with 15.25%) of the culturable microorganisms from genus *Pseudomonas*. The general reasons most probably were the increase of the amount of non-culturable microorganisms and also the enhanced role of the symbiotic and synergetic relationships in the biofilm when higher azo-dye concentrations were applied. The process of adaptation of given biological system towards aryl-containing xenobiotic biodegradation was already described by Topalova.[3] When the increasing concentration of the xenobiotic was applied to the complex biological systems (activated sludges or biofilms), the initial adaptive reaction towards biodegradation was on the basis of the increased amount of the target microorganisms (in our experiment this increase was with 48.76% compared with the beginning of the process). When the Amaranth concentration was increased, the amount of the culturable microorganisms from genus *Pseudomonas* in the biofilm was decreased with 15.25%. In the same time the biofilm increased the biodegradation activity and the rate of Amaranth removal was increased significantly. Then the symbiotic and synergetic relationships played significantly a more important role. In our study this was proved once again and it was also supported by data obtained on genetic level – it was shown that in the biofilm redistribution occurred in the location of the microorganisms from genus *Pseudomonas*. In the same time the zones with high concentration of bacteria from the target genus were formed. This suggested microstructuring within the biofilm and was accompanied with very high efficiency of the detoxification process, achieved when the xenobiotic pressure was strongest. The increased fluorescence signal during the late phase led to the conclusion of the increased amount of non-culturable microorganisms and also of increased metabolic activity of the bacteria from genus *Pseudomonas* (since the increased metabolic activity was associated with large amounts of rRNA which bonded a large quantity of the probe labelled with the fluorescent dye).

## Conclusion

The development of the biofilm microbial community was characterized on four levels in this study – technological, microbiological, enzymological and genetic levels. The genetic basis of the wastewater treatment process was reached by using, for the first time in Bulgaria for bacteria, the FISH. In this way the speculations for the development of detoxification potential of the complicated biological systems like biofilms towards aryl-xenobiotic biodegradation were proved and decoded on the genetic level in the strongest confirmation by efficiency of biodegradation and enzyme activities.

Technological parameters showed the increased xenobiotic biodegradation capacity of the biofilter. The activities of the key for the biodegradation enzymes supported evolution of the biodegradation potential by revealing the biochemical mechanisms of the observed effects. Microbiological parameters suggested the enhanced role of non-culturable bacteria from genus *Pseudomonas* and from azo-degrading complex as well as confirmed the increased significance of the symbiotic, synergetic and co-metabolic relationships in the biofilm development in the course of the azo-detoxification technology. FISH supported this at qualitative and quantitative levels. The discussed data visually, and in numbers, proved the increase of the key non-culturable microorganisms for the detoxification. Moreover, the microorganisms from genus *Pseudomonas* were grouped in well-defined zones which are probable ‘hot spots’ for the biodegradation on the basis of symbiotic, synergetic and co-metabolic relationships.

This complex diagnostics of the running detoxification processes in the course of the technologies, supported by the genetic analysis of the key microorganisms, can be applied like algorithms for management of the rate and efficiency of the biodegradation.

## References

[cit0001] Topalova Y (2009). Biological control and management of wastewater treatment.

[cit0002] Grekova-Vasileva M (2009). Bioalgorithms for management of wastewater treatment in textile industry. PhD thesis.

[cit0003] Topalova Y (2009). Biological control and management of wastewater treatment. DSc thesis.

[cit0004] Gilbride KA, Lee D-Y, Beaudette LA (2006). Molecular techniques in wastewater: understanding microbial communities, detecting pathogens, and real-time process control. J Microbiol Methods..

[cit0005] Sanz JL, Köchling T (2007). Molecular biology techniques used in wastewater treatment: an overview. Process Biochem..

[cit0006] Grekova-Vasileva M, Topalova Y (2009). Enzyme activities and shifts in microbial populations associated with activated sludge treatment of textile effluents. Biotechnol Biotechnol Equipment..

[cit0007] Bitton G (2005). Wastewater microbiology.

[cit0008] Nielsen H, Daims H, Lemmer H (2009). FISH handbook for biological wastewater treatment: identification and quantification of microorganisms in activated sludge and biofilms by FISH.

[cit0009] Mara D, Horan N (2003). Handbook of wastewater microbiology.

[cit0010] Moter A, Göbel UB (2000). Fluorescence in situ hybridization (FISH) for direct visualization of microorganisms. J Microbiol Methods.

[cit0011] Yemendzhiev H, Alexieva Z, Krastanov A (2009). Decolorization of synthetic dye reactive blue 4 by mycelial culture of white-rot Fungi Trametes versicolor 1. Biotechnol Biotechnol Equipment..

[cit0012] Kirilova M, Todorova Y, Topalova Y, Dimkov R Modeling of azo-degradation process with two immobilized biological systems.

[cit0013] APHA, AWWA, WEF (1989). Standard methods for the examination of water and wastewater.

[cit0014] Feist CF, Hegeman GD (1969). Phenol and benzoate metabolism by Pseudomonas putida: regulation of tangential pathways. J Bacteriol..

[cit0015] Topalova Y, Dimkov R, Manolov R (1994). Influence of aril-containing xenobiotics concentration on the oxygenase activities. Biotechnol Biotechnol Equipment..

[cit0016] Veeger C, Darvactanian DV, Zeylemaker WP, Colowick SP, Kaplan NO (1969). Succinate dehydrogenase. Methods in enzymology.

[cit0017] Zimmerman T, Kulla GH, Leisinger T (1982). Properties of purified Orange II azoreductase, the enzyme initiating azo dye degradation by Pseudomonas KF46. Eur J Biochem..

[cit0018] Willetts AJ, Cain RB (1972). Microbial metabolism of alkylbenzenesulphonates. Bacterial metabolism of undecylbenzene-p-sulphonate. Biochem J..

[cit0019] Farr DR, Cain RB (1968). Catechol oxygenase induction in Pseudomonas aeruginosa. J Bacteriol..

[cit0020] Kochetov TA (1974). Handbook of enzymology.

[cit0021] Schleifer K-H, Amann R, Ludwig W, Rothemund C, Springer N, Dorn S, Galli E, Silver S, Witholt B (1992). Nucleic acid probes for the identification and in situ detection of pseudomonads. Pseudomonas: molecular biology and biotechnology.

[cit0022] Loy A, Maixner F, Wagner M, Horn M (2007). probebase - an online resource for rRNA-targeted oligonucleotide probes: new features 2007. Nucleic Acids Res..

[cit0023] Daims H, Lücker S, Wagner M (2006). daime, a novel image analysis program for microbial ecology and biofilm research. Environ Microbiol..

